# Persistence of C-reactive protein increased levels and high disease activity are predictors of cardiovascular disease in patients with axial spondyloarthritis

**DOI:** 10.1038/s41598-022-11640-8

**Published:** 2022-05-07

**Authors:** Luca Navarini, Damiano Currado, Annalisa Marino, Stefano Di Donato, Alice Biaggi, Francesco Caso, Luisa Costa, Marco Tasso, Piero Ruscitti, Viktoriya Pavlych, Onorina Berardicurti, Antonio Ciancio, Ilenia Pantano, Federica Camarda, Maria Sole Chimenti, Arianna D’Antonio, Francesco Ursini, Addolorata Corrado, Francesco Paolo Cantatore, Roberto Perricone, Giuliana Guggino, Francesco Ciccia, Paola Cipriani, Raffaele Scarpa, Antonella Afeltra, Roberto Giacomelli

**Affiliations:** 1 Rheumatology, Immunology, and Clinical Medicine Research Unit, Department of Medicine, Campus Bio-Medico University of Rome, via Alvaro del Portillo 21, 00128 Rome, Italy; 2 Immunorheumatology Unit, Fondazione Policlinico Universitario Campus Bio-Medico, Rome, Italy; 3 Rheumatology Unit, Department of Clinical Medicine and Surgery, University Federico II of Naples, Naples, Italy; 4 Department of Biotechnological and Applied Clinical Sciences, University of L’Aquila, L’Aquila, Italy; 5 Rheumatology Section, Department of Clinical and Experimental Medicine, University of Campania “Luigi Vanvitelli”, Naples, Italy; 6 Rheumatology Section, Department of Internal Medicine, University of Palermo, Palermo, Italy; 7 Rheumatology, Allergology and Clinical Immunology, Department of “Medicina Dei Sistemi”, Tor Vergata University of Rome, Rome, Italy; 8 Medicine and Rheumatology Unit, IRCCS Istituto Ortopedico Rizzoli, Bologna, Italy; 9 Rheumatology Clinic, Department of Medical and Surgical Sciences, University of Foggia, Foggia, Italy

**Keywords:** Autoimmunity, Ankylosing spondylitis

## Abstract

An accurate prediction of cardiovascular (CV) risk in patients with Axial Spondyloarthritis (axSpA) is a strong unmet need, as CV risk algorithms poorly perform in these subjects. The aim of this study was to establish whether the persistence of high C-reactive protein (CRP) and high disease activity may be considered predictive factors of CVD in axSpA. 295 patients without personal history of CVD, were consecutively enrolled in this study. To evaluate the relationship between CV events occurrence (fatal and non-fatal) and the persistence of increased CRP levels, ASDAS (Ankylosing Spondylitis Disease Activity Score) > 2.1, and BASDAI (Bath Ankylosing Spondylitis Disease Activity) > 4 during the follow-up, univariable and multivariable Cox Proportional Hazard Models have been performed. During follow-up (we analyzed 10-years retrospective data), 23 patients had a CV event. Multivariable Cox Proportional Hazard Models showed a strong association between CV event and the persistency of increased CRP levels (namely, percentage of visits in which CRP levels were increased) (HR = 1.03; 95%CI 1.015–1.045; *p* < 0.001), of ASDAS > 2.1 (HR = 1.014, 95%CI 1.000–1.028, *p* = 0.047), and of BASDAI > 4 (HR 1.019, 95%CI 1.006–1.033, *p* = 0.006) during follow-up, after adjustment for age, sex, and diabetes. This study suggests that persistence of increased CRP levels and high disease activity may be considered biomarkers to identify those axSpA patients at higher risk of CVD. Innovative axSpA-specific CV risk score, including these variables, have to be developed.

## Introduction

Spondyloarthritis (SpA) is a heterogeneous group of chronic inflammatory arthropathies, mainly affecting the spine (axial SpA, axSpA) but also involving peripheral joints, entheses and extra-articular sites^1^. AxSpA group includes both non-radiographic axSpA (nr-axSpa) and ankylosing spondylitis (AS)^2^. AS is characterized by spinal inflammation, resulting in a limitation of spinal mobility associated with radiological evidence of structural damage of the sacroiliac (SI) joints and spine^3^. AxSpA deeply affects both physical function and quality of life of patients; moreover, it is well known that the presence of different comorbidities may significantly influence the prognosis of this disease^4,5^.

In axSpA, as well as in other types of inflammatory arthritis, an increased cardiovascular (CV) risk has been reported^6,7^; specifically, axSpA patients show a 20–40% increase of CV mortality, when compared to general population. An increased prevalence of traditional CV risk factors, inflammation, and potential adverse effects of drugs, especially nonsteroidal anti- inflammatory drugs (NSAIDs), also contribute to CV comorbidity^8–11^.

As far as AS is concerned, European League Against Rheumatism (EULAR) recommends that physicians carry out an annual assessment of CV risk^12^. An accurate prediction of CV risk is still a strong unmet need, and the early identification of high-risk patients is mandatory to improve the outcome^13,14^. Several CV risk algorithms have been proposed. The performance and calibration of these algorithms for calculating CV risk in Rheumatoid Arthritis (RA) is still matter of debate^15^. Different scores, including Framingham Risk Score (FRS), Systematic Coronary Risk Evaluation (HeartScore), Reynold’s Risk Score (RRS), and QRISK2, under-estimate the CV risk in RA patients^15–17^. For this reason, EULAR recommended a multiplication factor of 1.5, in RA and other inflammatory arthritis, except for QRISK2, QRISK3, and ASSIGN, already including an intrinsic multiplication factor for RA. Adapting the CV risk algorithms, according to EULAR recommendations, does not provide a significant improvement in discriminative ability, both in Psoriatic Arthritis (PsA) and in AS, when compared to general population^18–20^, showing the huge limitations of EULAR-adapted traditional CV risk algorithms, in patients with AS. In this setting, a Machine-Learning approach showed that the most important variable for assessing CVD risk was the baseline CRP levels^19^. It is well known that CRP is 1 out of the 5 variables included in the ASDAS (Ankylosing Spondylitis Disease Activity Score)^21^ activity score, reflecting the role that systemic inflammation plays in maintaining an active disease. On this basis, the aim of this study was to understand whether the persistence of higher CRP levels during the follow-up, ASDAS > 2.1 (active disease) and/or BASDAI (Bath Ankylosing Spondylitis Disease Activity Index)^21^ > 4 was significantly correlated with the increased CV risk of these patients, independently of other traditional risk factors, to confirm that an optimal control of disease activity may improve the outcome of these patients.

## Results

Data from 295 AxSpA patients were analyzed. During follow-up, 23 patients experienced a CV event: 10 cases of myocardial infarction, 4 cases of stable angina, 3 cases of stroke, 3 cases of TIA, 2 cases of HF; 1 fatal CV event was reported. Patient’s demography and characteristics are summarized in Table 1.

**Table 1:** Demographic and clinical characteristics of the patients.

	Entire axSpA population (n = 295)	axSpA without CV event (n = 272)	axSpA with CV event (n = 23)	*P*
Male (%)	65.4	65.4	65.2	0.98
Age (years)	47 (40–56)	46 (39–55.5)	58 (52–66)	** < 0.0001**
Diagnostic delay, months, median (25^th^-75^th^ Pctl)	24.4 (2–85.2)	24.4 (4.1–85.2)	6.1 (0–62.8)	0.06
CVD family history (%)	21	19	38.1	**0.042**
Smokers (%)	31.4	32.8	13.6	0.14
Atrial fibrillation (%)	1.4	1.1	31.3	0.2
Diabetes (%)	6.4	5.5	17.4	**0.026**
Stage 3–5 of chronic kidney disease (%)	1.4	1.1	4.4	0.2
Migraine (%)	13.2	13.2	13	0.98
Total cholesterol (mg/dl), median (25th–75th Pctl)	189 (168–212)	187 (166–212)	193 (170–211)	0.49
HDL cholesterol (mg/dl), median (25th–75th Pctl)	53 (45–63)	53 (45–62.5)	58 (42.5–70.5)	0.36
LDL cholesterol (mg/dl), median (25th–75th Pctl)	110 (92–130)	110.5 (92–130)	107 (90.5–128.5)	0.79
Systolic blood pressure (mmHg), median (25th–75th Pctl)	120 (120–130)	120 (120–130)	125 (120–140)	0.13
Diastolic blood pressure (mmHg), median (25th–75th Pctl)	80 (80–85)	80 (80–85)	82.5 (80–87.5)	**0.049**
Antihypertensive treatment (%)	25.4	22.4	60.9	** < 0.0001**
BMI, median (25th–75th Pctl)	25.7 (23.4–30.9)	25.6 (23.2–30.5)	28.3 (24.8–37.3)	0.67
Treatment with acetyl-salicylic acid (%)	6.5	3.7	39.1	** < 0.0001**
Treatment with statins (%)	6.8	5.5	21.7	**0.003**
Radiographic evidence of sacroiliitis (%)	58.7	58.9	57.1	0.9
Radiographic evidence of syndesmophytes precence (%)	35.2	35.6	42.9	0.4
MRI evidence of sacroiliitis (%)	70.7	72	57.1	0.2
MRI evidence of spine inflammation (%)	17.5	17.8	14.3	0.7
AS in according to New York criteria (%)	72.3	71.4	82.6	0.3
CRP (mg/l), median (25th–75th Pctl)	3.04 (1–6.09)	3 (1–5.09)	7.6 (5.04–13)	** < 0.0001**
ESR (mm/h), median (25th–75th Pctl)	14 (6–29)	13 (6–28)	24 (10–40)	0.06
BASDAI, median (25th–75th Pctl)	4.04 (2.1–6)	4 (2–6)	5.9 (4–6.8)	**0.01**
ASDAS, median (25th–75th Pctl)	2.1 (1.3–3)	2.1 (1.1–3)	3.2 (3–3.7)	**0.0004**
History of peripheral disease (%)	53.6	52.6	65.2	0.2
History of enthesitis (%)	19.7	18.4	36.4	**0.04**
History of dactylitis (%)	7.8	7.7	8.7	0.9
History of IBD (%)	74.2/25.8	73.4/26.6	82.6/17.4	0.3
History of uveitis (%)	83.3/16.7	84.1/15.9	73.9/26.1	0.2
Infliximab treatment (%)	20.2	20.8	14.3	0.5
Adalimumab treatment (%)	31.1	30.6	36.4	0.6
Etanercept treatment (%)	22.4	22.2	25	0.8
Golimumab treatment (%)	10	10	10	1
Certolizumab pegol treatment (%)	1.7	98.2/1.4	100/0	0.5
Secukinumab treatment (%)	10.1	10.6	5	0.4
Salazopyrin treatment (%)	18.1	18.1	18.2	0.9
NSAIDs continous use/on demand (%)	32.7/17.1	34/17.8	18.2/9.1	0.09

*axSpA* axial spondyloarthritis; *CV* cardiovascular; *Pctl* percentile; *CVD* cardiovascular disease; *BMI* body mass index; *MRI* magnetic resonance imaging; *AS* ankylosing spondylitis; *CRP* C-reactive protein; *ESR* erythrocyte sedimentation rate; *BASDAI* Bath Ankylosing Spondylitis Disease Activity Index; *ASDAS* Ankylosing Spondylitis Disease Activity Score; *IBD* inflammatory bowel disease; *NSAID* Nonsteroidal anti-inflammatory drug.

All the patients were Caucasian, with a large preponderance of male (65.4%) and a median age of 47 (40–56) years.

At baseline, patients showed a median BASDAI value of 4.04 (2.1–6) and ASDAS-CRP value of 2.1 (1.3–3). Total cholesterol median value was 189 (168–212) mg/dL, HDL cholesterol median value was 53 (45–63) mg/dL, LDL cholesterol median value was 110 (92–130) mg/dL, and BMI median value of 25.7 (23.4–30.9); finally, 6.4% of patients were diabetic.

Patients experiencing CV events during follow-up were significantly older than patients without CV involvement (58 (52–66) vs. 46 (39–55.5) respectively, *p* < 0.0001). Furthermore, comparing patients experiencing CV events with the other patients, we observed that the former, at baseline, showed: i. higher prevalence of CVD family history (38.1% vs. 19%, respectively, *p* = 0.042), ii. higher prevalence of diabetes (17.4% vs. 5.5%, respectively, *p* = 0.026), iii. higher prevalence of enthesitis (36.4% vs. 18.4%, respectively, *p* = 0.04), iv. higher CRP levels (7.6 (5.04–13) vs. 3 (1–5.09) mg/dL respectively, *p* < 0.0001), v. higher diastolic blood pressure (82 (80–87.5) vs. 80 (80–85) mmHg, respectively, *p* = 0.049), vi. higher prevalence of anti-hypertensive treatment (60.9% vs. 22.4%, respectively, *p* < 0.0001), vii. higher prevalence of anti-platelets treatment (39.1% vs. 3.7%, respectively, *p* < 0.0001), viii. higher prevalence of statin treatment (21.7% vs. 5.5%, respectively, *p* = 0.003).

Finally, we must point out that patients experiencing CV events were affected from a more severe AxSpA, as showed by their higher BASDAI score and ASDAS-CRP score, when compared to the other patients (mean BASDAI 5.9 (4–6.8) vs. 4 (2–6), respectively, *p* = 0.01; mean ASDAS-CRP:3.2 (3–3.7) vs. 2.1 (1.1–3), respectively, *p* = 0.0004).

A close correlation between the occurrence of CV events and the increased CV risk prediction scores, assessed according to the original algorithms and according to the EULAR-adapted algorithms, was observed, as reported in Table 2.

**Table 2:** CV risk algorithms of the patients with axSpA.

	Entire axSpA population (n = 295)	axSpA without CV event (n = 272)	axSpA with CV event (n = 23)	*P*
CUORE	2.2 (0.9–6)	2 (0.9–5.4)	4.95 (1.7–12.7)	**0.01**
CUORE*1.5	3.3 (1.35–9)	3 (1.35–8.1)	7.425 (2.55–19.05)	**0.01**
FRS	6.8 (3.8–15.6)	6.5 (3.5–15)	13.35 (6.7–23.8)	**0.02**
FRS*1.5	10.2 (5.7–23.4)	9.75 (5.25–22.5)	20.025 (10.05–35.7)	**0.02**
QRISK2	4.1 (1.7–12.3)	3.65 (1.5–10.7)	12.5 (5.8–22.3)	**0.002**
QRISK2-RA	5.25 (2.25–15.65)	4.75 (2–13.7)	16.16 (7.6–28)	**0.002**
QRISK3	3.7 (1.5–10.9)	3.3 (1.4–10.1)	12.25 (5–20)	**0.002**
QRISK3-RA	4.6 (1.9–13.25)	4.1 (1.7–12.3)	14.95 (6.1–24)	**0.002**
RRS	3 (2–7)	2.5 (2–7)	6.5 (4–12)	**0.006**
RRS*1.5	4.5 (3–10.5)	3.75 (3–10.5)	9.75 (6–18)	**0.006**
ASSIGN	6 (4–13)	6 (3–13)	13 (6–28)	**0.006**
ASSIGN-RA	8 (4–16)	7 (4–16)	16.5 (7–35)	**0.004**
HeartScore	1 (1–2)	1 (1–2)	1 (1–4)	**0.047**
HeartScore*1.5	1.5 (1.5–3)	1.5 (1.5–3)	1.5 (1.5–6)	**0.047**

*axSpA* axial spondyloarthritis; *FRS* Framigham risk score; *RA* rheumatoid arthritis; *RRS* Reynolds risk score; *Score* Systematic Coronary Risk Evaluation.

Table 3 shows Univariable Cox Proportional Hazard Model. We observed a significant association between the incidence of CV events and these variables at baseline: i. older age (HZ = 1.084, 95% CI 1.04–1.129; *p* < 0.001), ii. prevalence of diabetes (HZ = 3.137, 95% CI 1.053–9.344; *p* = 0.04), iii. baseline HDL cholesterol values (HZ = 1.035; 95% CI 1.006–1.064; *p* = 0.016), iv. presence of enthesitis (HZ = 2.901, 95% CI 1.167–7.21; *p* = 0.022), v. presence of uveitis (HZ = 6.596, 95% CI 1.902–22.879, *p* = 0.003). vi. hypertension treatment (HZ = 5.093, 95% CI 2.048–12.66; *p* < 0.001), vii. anti-platelet treatment (HZ = 10.135, 95% CI 4.265–24.083; *p* < 0.001), viii. statin treatment (HZ = 3.229, 95% CI 1.176–8.866; *p* = 0.023). Of interest, as far as the variables regarding the activity of the disease are concerned, a significant association between the occurrence of CV events and: i. CRP value at baseline (HZ = 4.666, 95% CI 1.702–12.788; *p* = 0.003); ii. the persistence of CRP levels above the normal values (> 5 mg/L) (HZ = 1.033, 95% CI 1.019–1.047, *p* < 0.001); iii.the percentage of time, during follow-up, in which ASDAS > 2.1 (HZ = 1.016, 95% CI 1.003–1.028, *p* = 0.012); iv. ASDAS-CRP score at baseline (HZ = 1.624, 95% CI 1.053–2.506, *p* = 0.028) was observed.

**Table 3:** Univariable Cox Proportional Hazard Models considering as dependent variable the occurrence of the first CV event during follow-up.

	HR	95%CI	*p*
Time percentage of elevated CRP (> 5 mg/l) during follow-up	1.033	1.019–1.047	** < 0.001**
Time percentage of ASDAS > 2.1 during follow-up	1.016	1.003–1.028	**0.012**
Time percentage of BASDAI > 4 during follow-up	1.013	1.001–1.025	**0.03**
Age at the enrollment	1.084	1.04–1.129	** < 0.001**
Diabetes	3.137	1.053–9.344	**0.04**
HDL cholesterol	1.035	1.006–1.064	**0.016**
Antihypertensive treatment	5.093	2.048–12.66	** < 0.001**
Acetyl-salicylic acid treatment	10.135	4.265–24.083	** < 0.001**
Statins treatment	3.229	1.176–8.866	**0.023**
Increased levels of CRP (> 5 mg/l) at the enrollment	4.666	1.702–12.789	**0.003**
ASDAS at the enrollment	1.624	1.053–2.506	**0.028**
Enthesitis at the enrollment	2.901	1.167–7.21	**0.022**
Uveitis at the enrollment	6.596	1.902–22.879	**0.003**
Male sex	1.272	0.526–3.074	0.593
CVD family history	1.94	0.79–4.764	0.149
Smoking habit	0.636	0.301–1.34	0.236
Total Cholesterol	0.999	0.989–1.011	0.997
LDL Cholesterol	0.991	0.975–1.007	0.261
Systolic blood pressure	1.02	0.99–1.051	0.187
Diastolic blood pressure	1.033	0.998–1.07	0.066
BMI	1	0.999–1	0.095
Increased levels of ESR (mm/h) at the enrollment	1.44	0.623–3.326	0.393
BASDAI	1.187	0.955–1.476	0.122
Peripheral Disease at the enrollment	1.565	0.661–3.707	0.309
History of peripheral disease	1.679	0.7015–4.016	0.245
History of enthesitis	1.803	0.717–4.536	0.210
Dactylitis at the enrollment	1.307	0.175–9.746	0.794
History of dactylitis	.423	0.053–3.395	0.418
Psoriasis at the enrollment	2.866	0.962–8.541	0.059
History of psoriasis	1.897	0.637–5.645	0.250
IBD at the enrollment	0.876	0.2915–2.633	0.814
History of uveitis	1.922	0.745–4.954	0.176
Increased levels of Total cholesterol at the enrollment	0.672	0.287–1.575	0.361
Increased levels of HDL cholesterol at the enrollment	1.076	0.453–2.559	0.868
Increased levels of LDL cholesterol at the enrollment	0.456	0.186–1.121	0.087

*HR* hazard ratio; *CI* confidence interval; *BMI* body mass index; *CRP* C-reactive protein; *ESR* erythrocyte sedimentation rate; *BASDAI* Bath Ankylosing Spondylitis Disease Activity Index; *ASDAS* Ankylosing Spondylitis Disease Activity Score; *IBD* inflammatory bowel disease.

Due to the low number of CV events reported during follow-up, a non-parsimonious approach in multivariable statistical analysis was not possible. Nevertheless, different Multivariable Cox Proportional Hazard Models confirmed a strong association between CV events and: i. the persistency of increased CRP levels during the follow-up; ii. the persistency of high or very high disease activity, as shown by ASDAS-CRP scores; iii. the persistency of high disease activity according to BASDAI, as reported, in detail in Table 4. Due the substantial collinearity between the duration of elevated CRP and the duration of elevated ASDAS-CRP, only a multivariable model comprising age, gender, duration of elevated CRP, and duration of BASDAI > 4 was performed, showing that the duration of elevated CRP and the duration of BASDAI > 4 were significantly associated with CV events (HZ 1.032, 95%CI 1.019–1.046, *p* < 0.001 and HZ 1.013, 95%CI 1.001–1.026, *p* = 0.04, respectively).

**Table 4:** Multivariable Cox Proportional Hazard Models considering as dependent variable the occurrence of the first CV event during follow-up.

	Model 1 (corrected for age and gender)		Model 2 (corrected for age, gender, and diabetes)		Model 3 (corrected for age, gender, acetyl-salicylic acid and statin treatments)		Model 4 (corrected for age, gender, and anti-hypertensive treatment)	
	HR (95%CI)	*p*	HR (95%CI)	*p*	HR (95%CI)	*p*	HR (95%CI)	*p*
Time percentage of elevated CRP during follow-up	1.028 (1.013–1.042)	** < 0.001**	1.030 (1.015–1.045)	** < 0.001**	1.027 (1.012–1.042)	** < 0.001**	1.027 (1.12–1.042)	** < 0.001**
Time percentage of ASDAS-CRP > 2.1 during follow-up	1.014 (1.001–1.027)	**0.03**	1.014 (1.000- 1.028)	**0.047**	1.013 (1.000–1.027)	**0.05**	1.013 (1.001–1.026)	**0.037**
Time percentage of BASDAI > 4 during follow-up	1.019 (1.006–1.033)	**0.004**	1.019 (1.006–1.033)	**0.006**	1.021 (1.006–1.035)	**0.005**	1.018 (1.004–1.031)	**0.01**

Each independent variable (time percentage of elevated CRP during follow-up; time percentage of ASDAS-CRP > 2.1 during follow-up; or time percentage of BASDAI > 4 during follow-up) was tested in four models: model 1, corrected for age and gender; model 2, corrected for age, gender, and diabetes; model 3, corrected for age, gender, acetyl-salicylic acid and statin treatments; model 4, corrected for age, gender, and anti-hypertensive treatment. *HR* hazard ratio; *CI* confidence interval; *CRP* C-reactive protein; *ESR* erythrocyte sedimentation rate; *BASDAI* Bath Ankylosing Spondylitis Disease Activity Index; *ASDAS* Ankylosing Spondylitis Disease Activity Score.

## Discussion

CVD repreents one of the more important causes of morbidity and mortality in patients with axSpA^22^. An accurate prediction of CV risk may lead to the development of preventive strategies to improve the overall outcome^23^.

In this study, we analyzed 10-years retrospective data of a large multicenter cohort of axSpA patients, to provide a real-life estimation of the CV burden. At the best of our knowledge, our cohort is one the largest cohorts published in available literature, and this large number of patients partially overcomes the limitations of a retrospective design. Here, we showed that both a persistent high CRP values and/or persistent high disease activity may be considered very sensitive biomarkers of CV events, in patients with axSpA.

Patients experiencing CV events during follow-up significantly differ from the patients without CV complications. CV events mainly affected older patients and patients with higher prevalence of traditional CV risk factors, such as higher prevalence of CVD family history, diabetes, anti-hypertensive, acetylsalicylic acid and statin treatment, diastolic blood pressure, compared to patients without CV events.

Despite of the well-known role of traditional CV risk factors, to induce CV events in general population, our analysis failed to show any possible association among some of these factors and the development of CV events, in axSpA^24,25^. As far as hypercholesterolemia and BMI are concerned, we did not find any association with the insurgence of new CV events. It is well known that in inflammatory arthritis the so called “lipid paradox”^26^, modifying the composition of lipids, during inflammation, impairs the predictive role of BMI and lipids profile on CVD. although this paradox has been largely studied in RA, it is a matter of debate if the systemic inflammatory process, observed in the other inflammatory joint diseases, may induce similar effects^27^. Our study, mirroring what observed in RA, suggest a negative predictive role of lipids concentration on CVD, also in axSpA, whereas this result needs to be confirmed in studies specifically designed. We must point out that axSpA patients experiencing CV events during follow-up showed higher disease activity at the first visit, as confirmed by higher CRP, BASDAI and ASDAS-CRP levels. These data confirm the strong association between systemic chronic inflammation and CV comorbidity and mortality. We may also suggest that the occurrence of fatal or non-fatal CV events may derive from a synergy between both axSpA-related factors and traditional CV risk factors, confirming the need of a strict management of systemic inflammation to improve the long-term outcome of axSpA.

These data confirm the results of our previous work, showing the lower effect of the traditional CV risk factors, used by FRS and other traditional CV risk algorithms, in predicting the development of CV events in patients with axSpA and suggesting the huge limitations of both traditional and EULAR-adapted CV risk algorithms in these patients^18^.

Furthermore, we were able to show that not only increased CRP and higher disease activity scores at the first visit but also their persistence during the follow-up may be predictive markers of CV events in our patients. In fact, as confirmed by the univariable model, patients experiencing fatal or non-fatal CV events had persistently both higher CRP value and higher disease activity scores.

These data were furtherly confirmed by the multivariable Cox Proportional Hazard Model, highlighting that a persistent high CRP value, as well as a persistent high disease activity score (ASDAS > 2.1 or BASDAI > 4), independent of the patient's age, gender, and main metabolic comorbidities, are strongly correlated with the occurrence of a new fatal or non-fatal CV event.

Taken together, our data strongly suggest the role of the inflammatory process in those axSpA patients with a persistent, poorly controlled, active disease in increasing the risk of CV events.

We are aware of some possible limitations of our study, which did not allow us to fully capture the great heterogeneity of CV risk in axSpA population, despite of the large number of patients enrolled, considering the relative rarity of the disease. First, a non-parsimonious approach to multivariable analysis has been used due to the relatively low number of CV events reported during follow-up. Moreover, due to the observational design of this study, it could be subjected to a number of possible biases. We tried to minimize the main methodological problems by a careful definition of each variable to be assessed. Furthermore, patients with significant missing data, which were considered meaningful for the analyses, were removed. Specifically, patients with missing data in the main outcomes were removed from the analyses. On the other hand, our study would provide a “real-life” estimation of the occurrence of CV events in consecutive patients with axSpA, admitted in 8 Italian Rheumatology Units. Moreover, analyzing a real-life cohort, in which the treatments of axSpA patients were not randomized, we did not analyze any possible association between the effect of anti-rheumatic treatments and the CV outcomes, thus avoiding the risk of a “confounding by indication” bias, a bias deriving when physicians decided to prescribe a more intensive treatment to those patients that, in their opinion, are affected by a more aggressive disease [22]. In this context, the lack of RCTs, specifically designed to evaluate the effect of different drugs in controlling the insurgence of CVD, strongly limits the possibility to reach robust conclusions.

On the other hand, despite the reported limitations, this study strongly confirms, in a very large cohort of patients, that persistent elevation of CRP levels and disease activity scores during follow-up may be considered the most important biomarkers to identify axSpA patients at higher risk of CVD. These results could lead the rheumatologic scientific community to develop a CV risk prediction algorithm in which CRP and disease activity scores may have a more relevant weight, to earlier identify those patients with an increased CV risk, thus implementing preventive strategies to modify the overall outcome of these patients.

## Methods

A retrospective analysis of data from AxSpA cohorts of 8 Italian Rheumatology Units has been performed, in accordance with the STROBE guidelines. The observational time frame was 2010–2020, and 295 patients, fulfilling the 2009 ASAS (Assessment of Spondyloarthritis International Society) Criteria^28^, without a history of CVD before 2010, were consecutively included. Although unequal follow-up time was allowed [the median duration in our cohort was 42 months (24–84)], two visits/year had to be done.

The study was approved by the Ethics Committee of University of Rome “Campus Bio-Medico" (approval number: 60/18 OSS), and conducted according to the Declaration of Helsinki and its amendments. Written informed consent was obtained from all patients. All patients received one visit/semester. Baseline characteristics included: age, gender, weight (kg), height, CRP (mg/L), erythrocyte sedimentation rate (ESR) (mm/h), axial arthritis (grade of radiographic sacroiliitis: 0, I, II, III, IV; non-radiographic sacroiliitis), peripheral arthritis, Bath Ankylosing Spondylitis Disease Activity Index (BASDAI), Bath Ankylosing Spondylitis Functional Index (BASFI)^21^, enthesitis, dactylitis, psoriasis, history of inflammatory bowel diseases (IBD), history of uveitis, family history of CVD, smoking status, hypertension, use of antihypertensive medication, use of statins and aspirin, diabetes mellitus, atrial fibrillation, chronic kidney disease stage IV–V, angina or heart attack in a 1st-degree-relative\60 years, systolic blood pressure (SBP), total cholesterol, and high-density lipoprotein cholesterol (HDL-C).

CV events (fatal and non-fatal) included in our database were: sudden cardiac death, coronary artery diseases (CAD) (stable and unstable angina, myocardial infarction), cerebral vascular accident (CVA), transient ischemic attack (TIA), peripheral artery disease (PAD) and heart failure (HF). The baseline 10-year general FRS for CVD, QRISK2, QRISK3, CUORE, RRS, and ASSIGN were calculated using already-published algorithms^15–18,29,30^. SCORE algorithm for low-risk countries was used^18,31^. The default median value 15.89 for the Scottish Index of Multiple Deprivation (SIMD) was used to calculate the ASSIGN score. Two patients were excluded from the analysis due to missing data regarding the main outcomes.

Continuous variables are reported as median (25th-75th percentile), while categorical variables are reported as percentage. Chi2 test was used for analysis of contingency tables, while Mann–Whitney test was used to compare ranks; to evaluate the relationship between CV events and time duration of increased CRP levels (percentage of follow-up visits with CRP levels > 5 mg/L), high or very high disease activity according to ASDAS-CRP (percentage of follow-up visits with ASDAS-CRP > 2.1), and/or high disease activity according to BASDAI (percentage of follow-up visits with BASDAI > 4) univariable and multivariable Cox Proportional Hazard Models were performed. Stata V.14 was used for statistical analysis.
